# Supplementary figures and images for: A time-varying geospatial model of habitat suitability for Japanese encephalitis virus vectors and vertebrate hosts in Australia
Source: PLoS Negl Trop Dis. 2026 Mar 20;20(3):e0014127. doi: 10.1371/journal.pntd.0014127 (PMC13035343; doi:10.1371/journal.pntd.0014127)

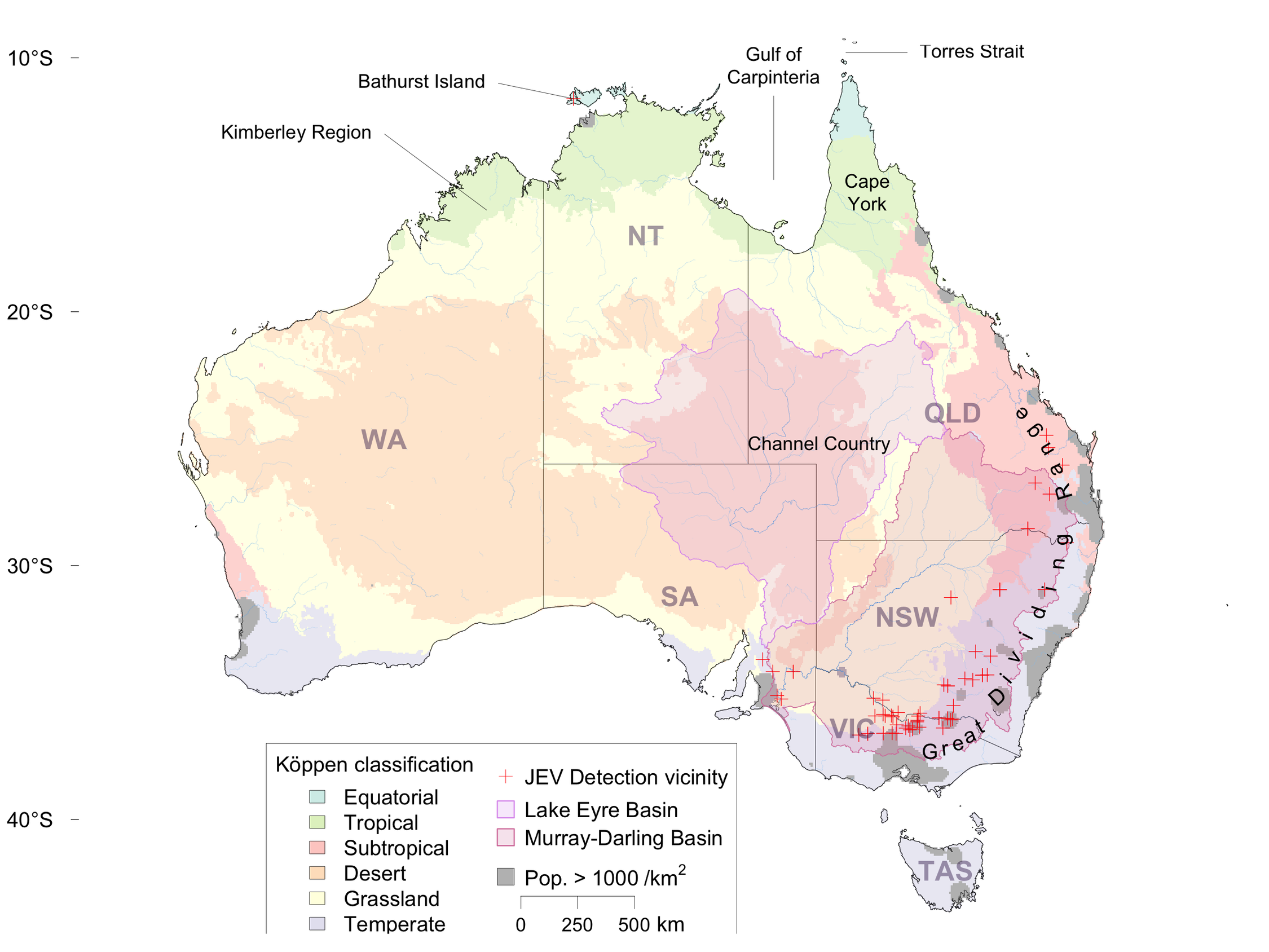

Supplement: S1 Fig — Base maps obtained from Australian Bureau of Statistics (CC BY 4.0) https://www.abs.gov.au/statistics/standards/australian-statistical-geography-standard-asgs-edition-3/jul2021-jun2026/access-and-downloads/digital-boundary-files. (TIF) [file pntd.0014127.s001.tif]

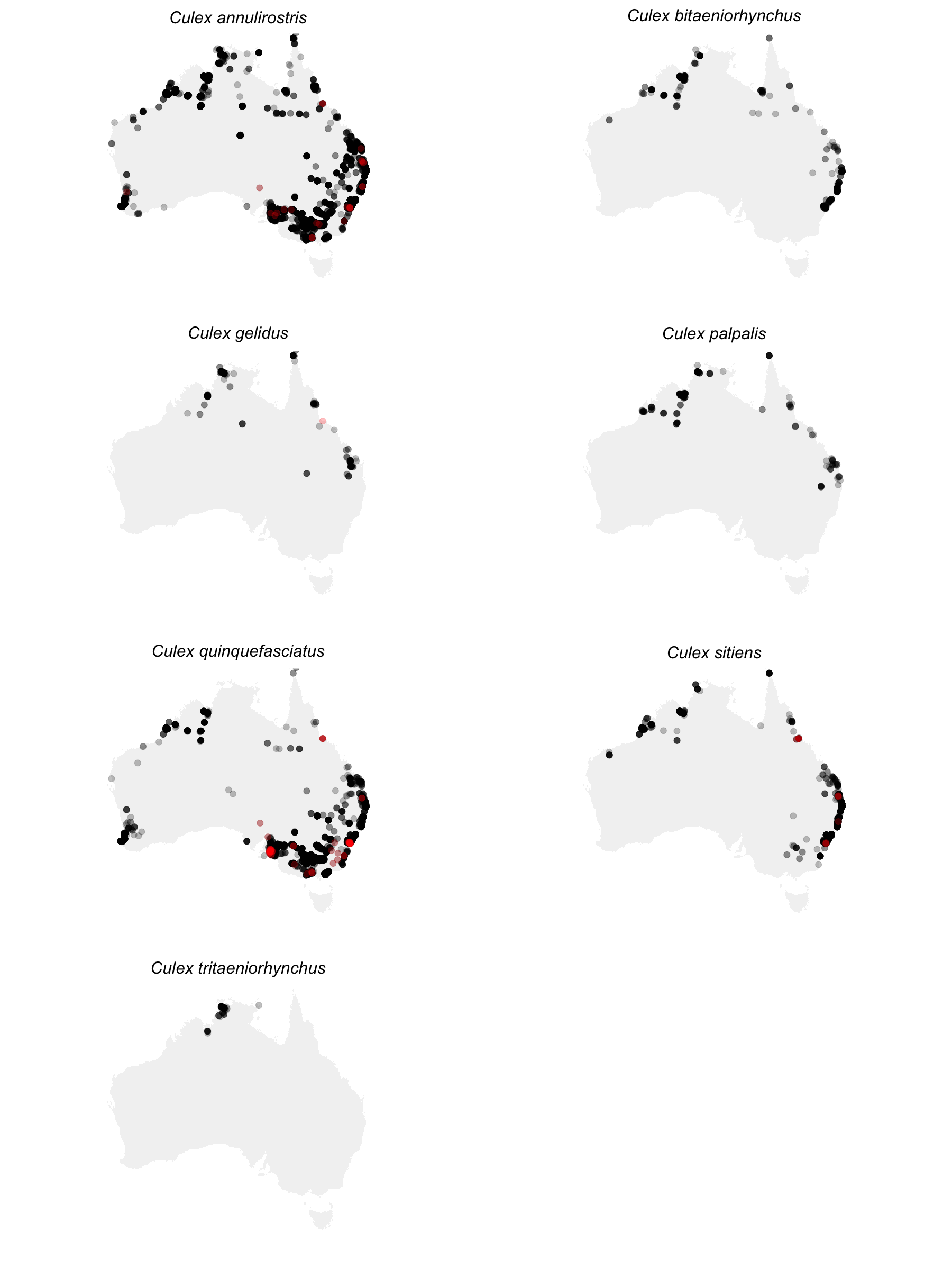

Supplement: S2 Fig — Black points indicate records obtained from Australian state and territory surveillance and published literature and databases. Red points were obtained from Global Biodiversity Information Facility and Atlas of Living Australia. Points are semi-transparent (alpha = 0.25). Those that appear solid result from accumulation of at least four records at one location. Base maps obtained from Australian Bureau of Statistics (CC BY 4.0) https://www.abs.gov.au/statistics/standards/australian-statistical-geography-standard-asgs-edition-3/jul2021-jun2026/access-and-downloads/digital-boundary-files. (TIF) [file pntd.0014127.s002.tif]

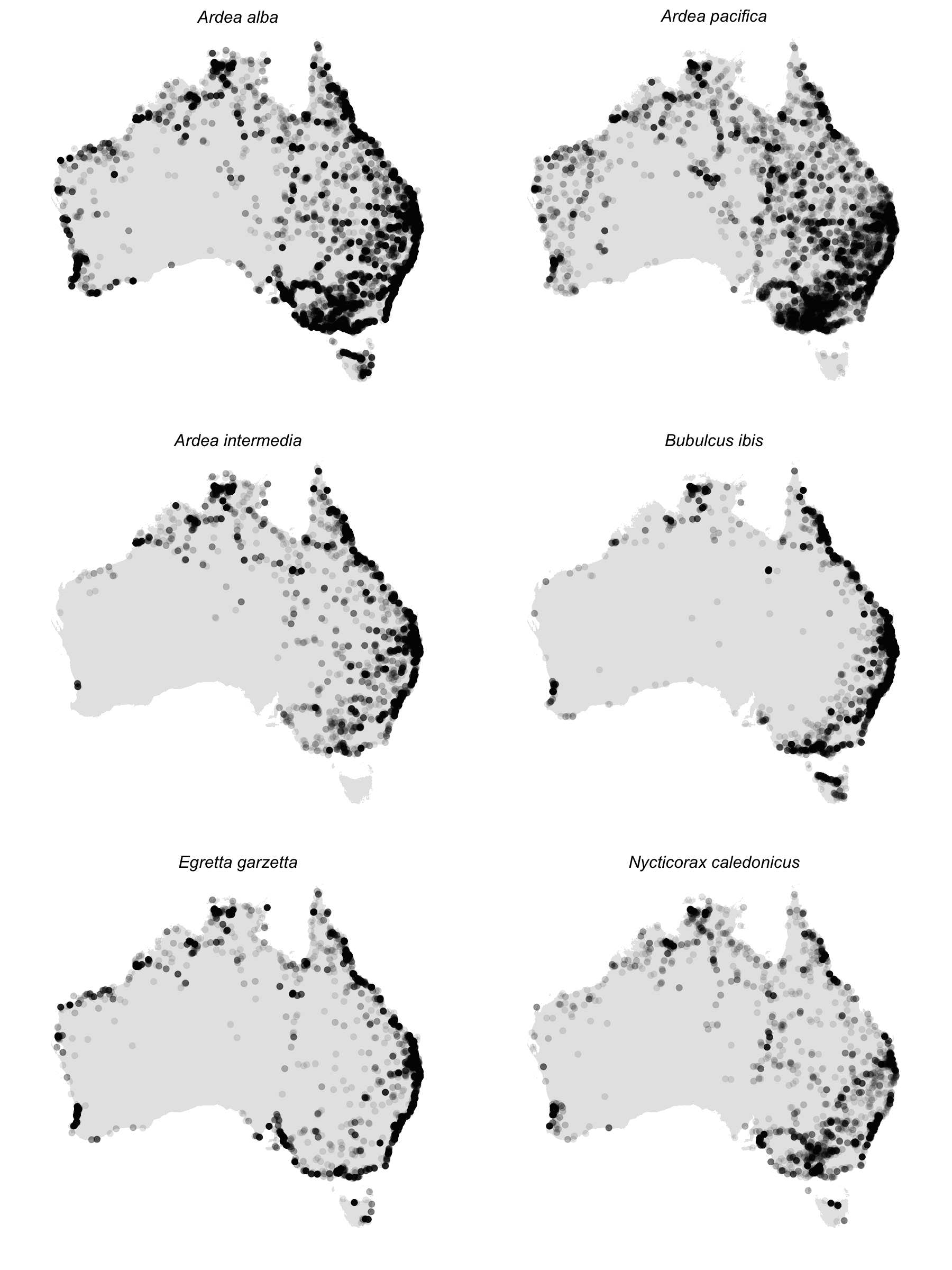

Supplement: S3 Fig — Points are semi-transparent (alpha = 0.1). Those that appear solid result from accumulation of at least 10 records at one location. Base maps obtained from Australian Bureau of Statistics (CC BY 4.0) https://www.abs.gov.au/statistics/standards/australian-statistical-geography-standard-asgs-edition-3/jul2021-jun2026/access-and-downloads/digital-boundary-files. (TIF) [file pntd.0014127.s003.tif]

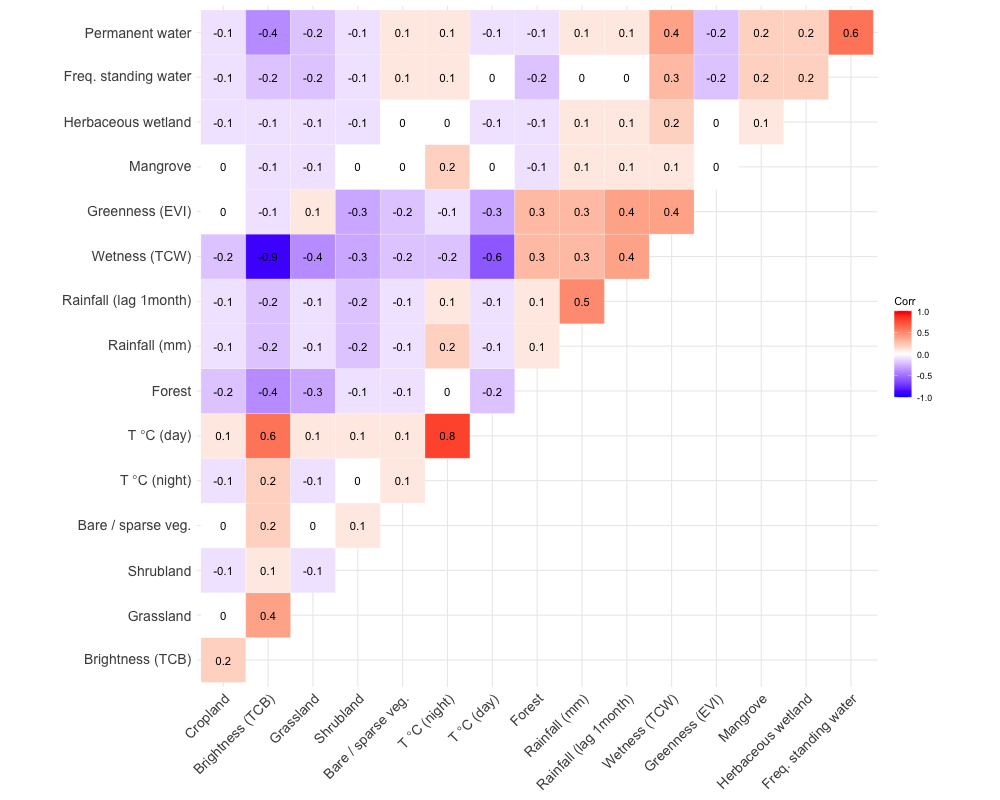

Supplement: S4 Fig — (TIF) [file pntd.0014127.s004.tif]

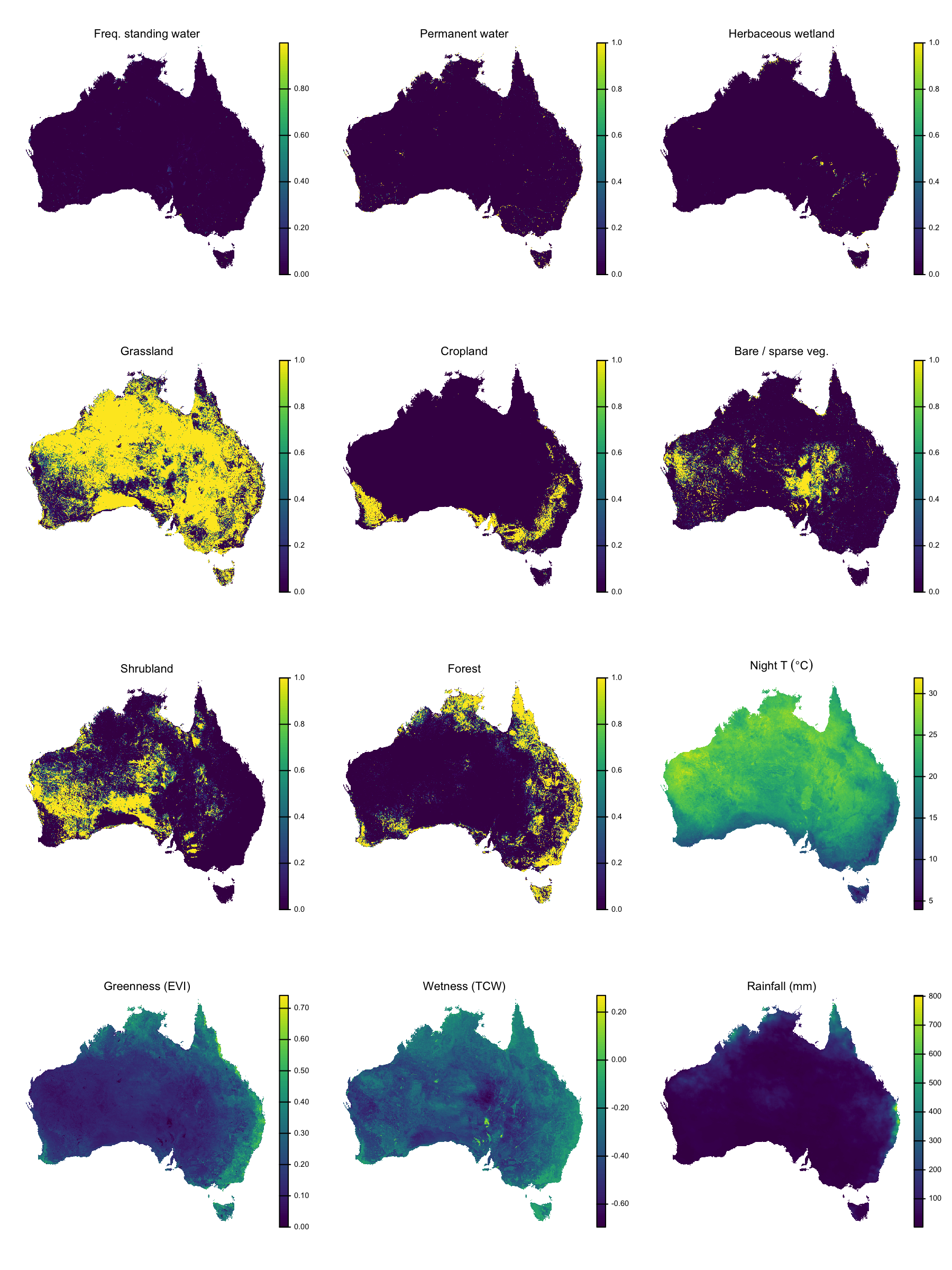

Supplement: S5 Fig — The values correspond to a 1 km grid. Values for February 2022 are displayed for the temporally dynamic environmental variables (Night T C, Greenness (EVI), Wetness (TCW) and Rainfall (mm). Base maps obtained from Australian Bureau of Statistics (CC BY 4.0) https://www.abs.gov.au/statistics/standards/australian-statistical-geography-standard-asgs-edition-3/jul2021-jun2026/access-and-downloads/digital-boundary-files. (TIF) [file pntd.0014127.s005.tif]

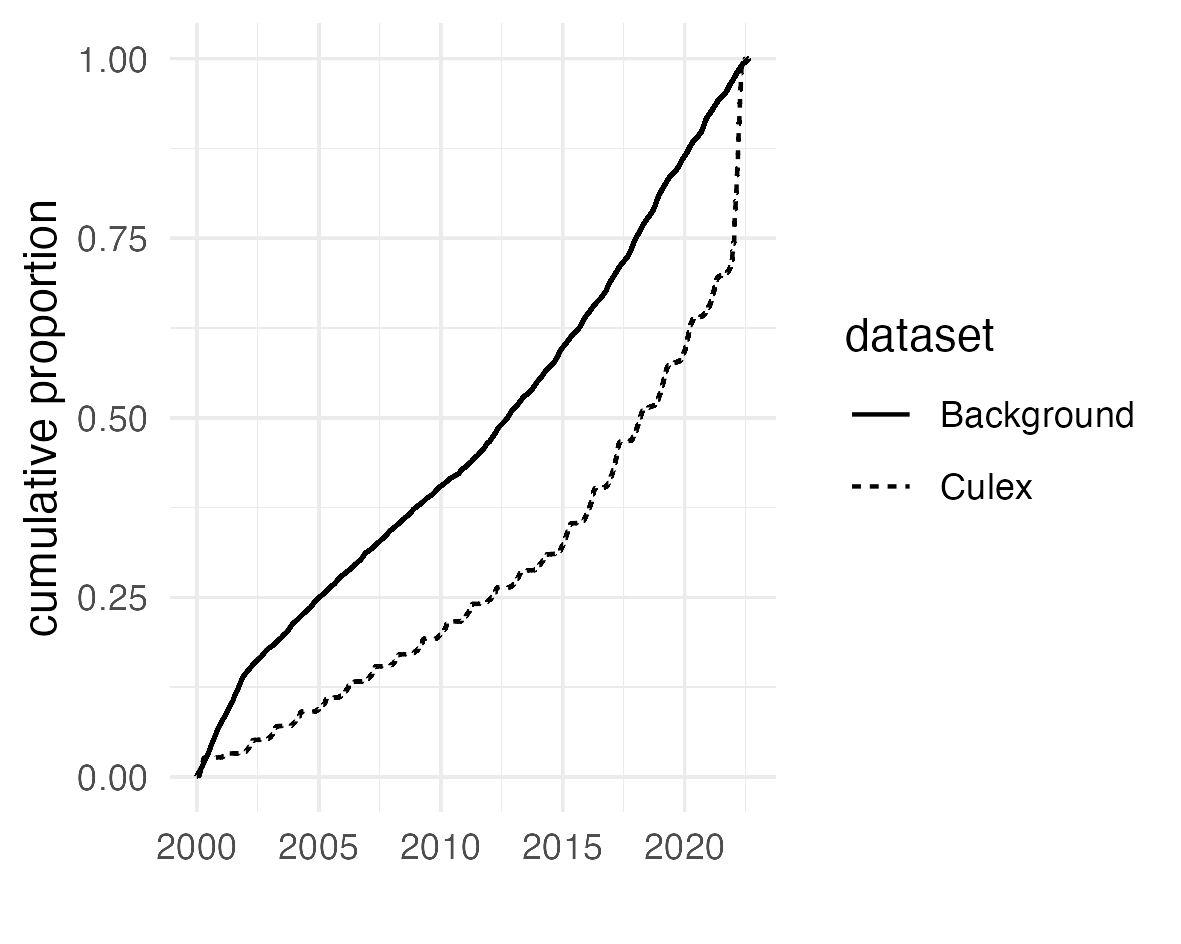

Supplement: S6 Fig — (TIF) [file pntd.0014127.s006.tif]

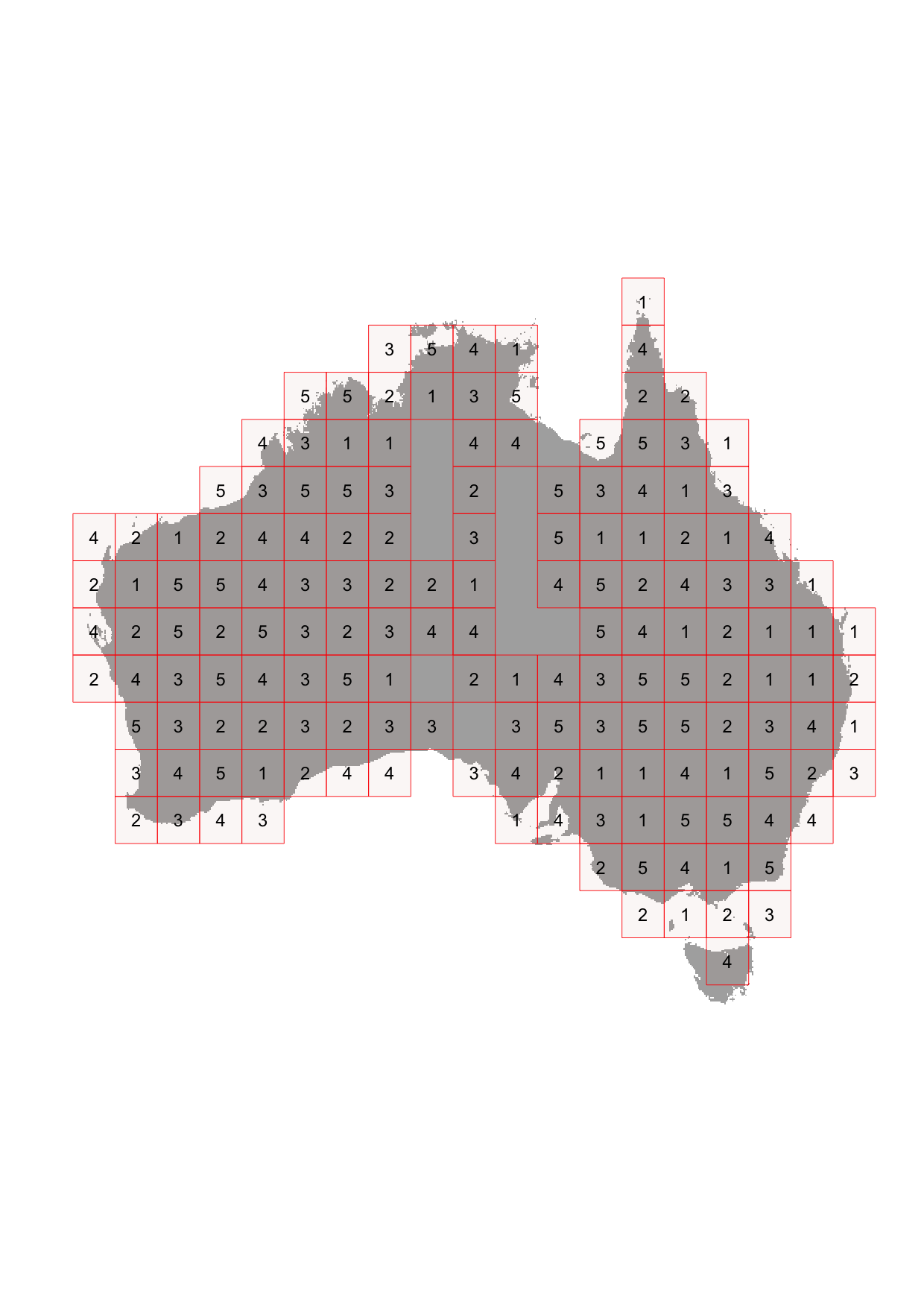

Supplement: S7 Fig — Each block is labelled with the cross-validation fold to which it was randomly assigned subject to the overall criterion of approximate balance of presence and target background points per fold. Base maps obtained from Australian Bureau of Statistics (CC BY 4.0) https://www.abs.gov.au/statistics/standards/australian-statistical-geography-standard-asgs-edition-3/jul2021-jun2026/access-and-downloads/digital-boundary-files. (TIF) [file pntd.0014127.s007.tif]

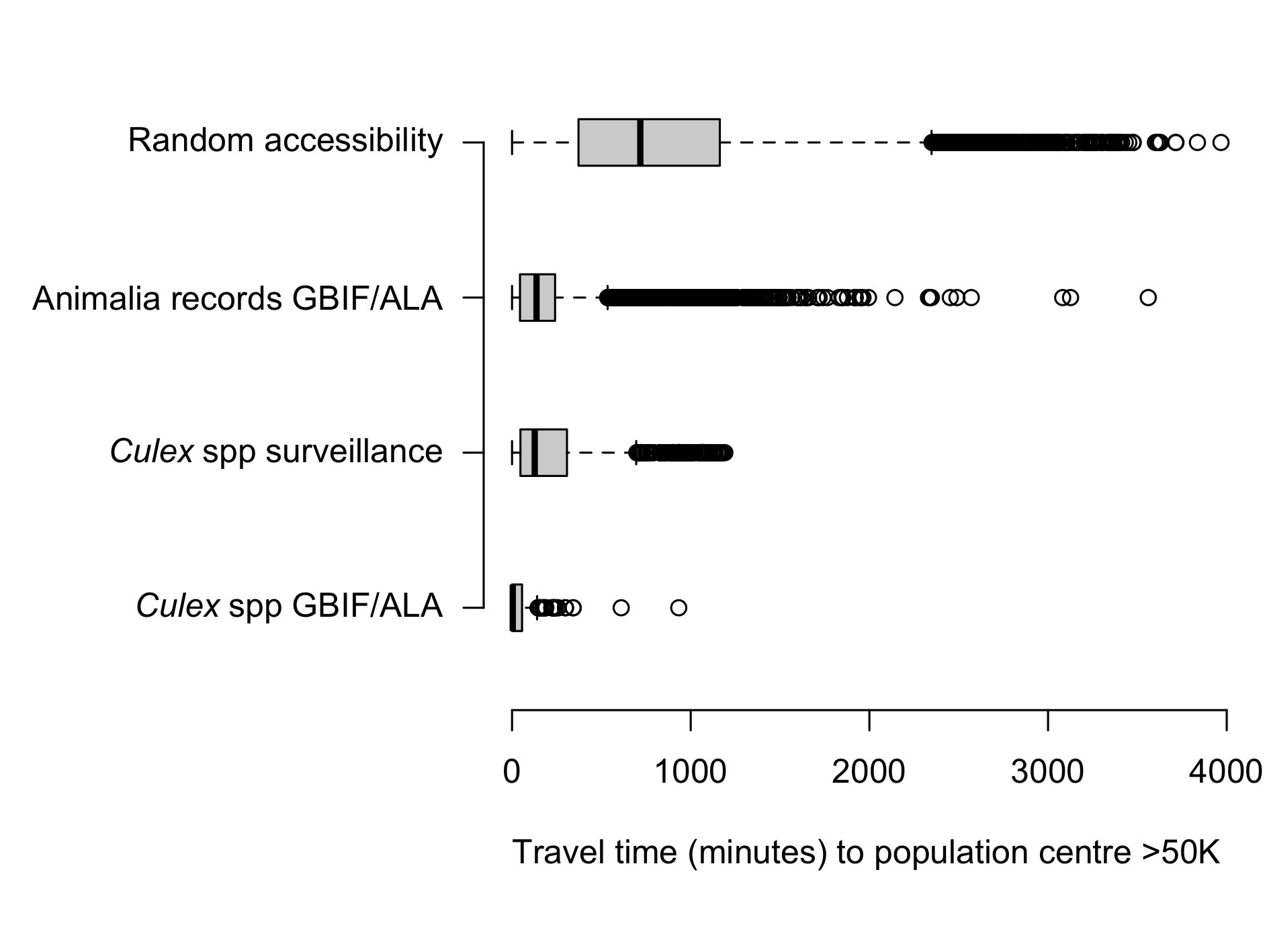

Supplement: S8 Fig — (TIF) [file pntd.0014127.s008.tif]

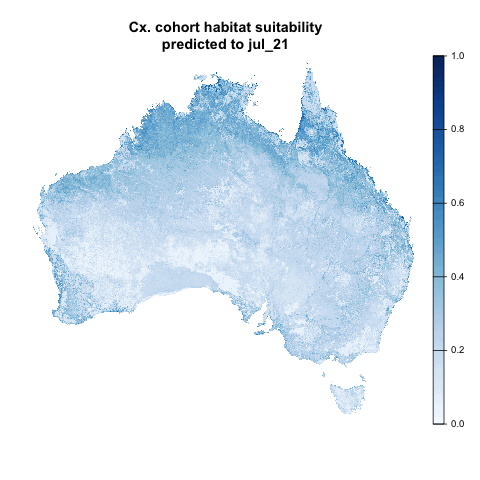

Supplement: S1 File — Monthly predictions of habitat suitability for Culex cohort to July 2021–June 2023 available at this link. Base maps obtained from Australian Bureau of Statistics (CC BY 4.0) https://www.abs.gov.au/statistics/standards/australian-statistical-geography-standard-asgs-edition-3/jul2021-jun2026/access-and-downloads/digital-boundary-files. (GIF) [file pntd.0014127.s009.gif]

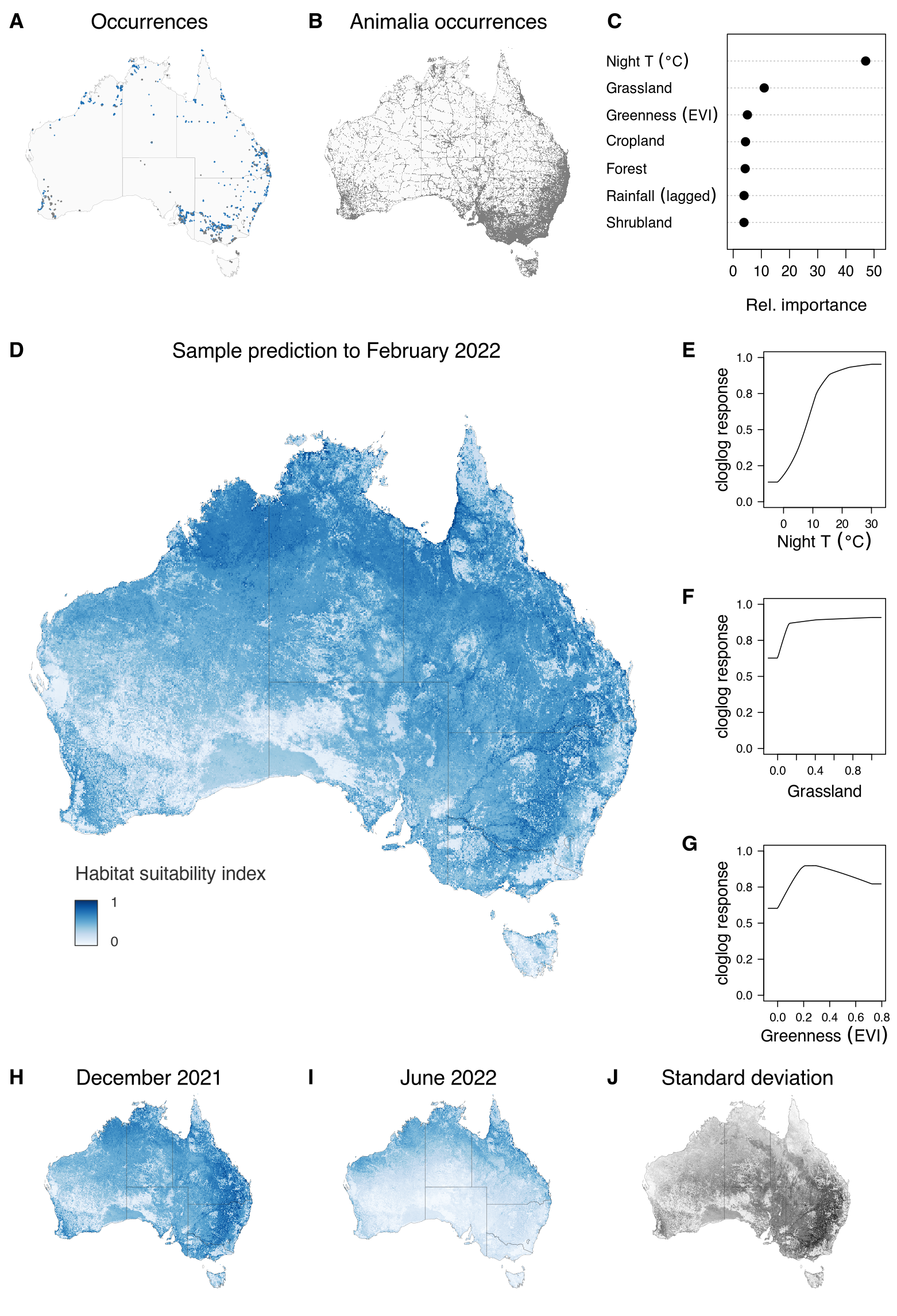

Supplement: S9 Fig — A) the distribution of the combined occurrence data for Culex annulirostris; B) the distribution of background occurrence data for Kingdom Animalia, including any trap sites where other mosquitoes but not Cx annulirostris were trapped; C) the relative ‘importance’ of top seven covariates as calculated by permutation on ensemble fit; D) prediction of relative habitat suitability to the outbreak period (February 2022); E–G) the shape of the marginal relationship between relative habitat suitability and the three most influential predictor variables; H–J) prediction of relative habitat suitability from December 2021 and June 2022, and standard deviation of predictions across the temporal sequence from July 2021 to June 2023. Base maps for A, B, and H–J obtained from Australian Bureau of Statistics (CC BY 4.0) https://www.abs.gov.au/statistics/standards/australian-statistical-geography-standard-asgs-edition-3/jul2021-jun2026/access-and-downloads/digital-boundary-files. (TIF) [file pntd.0014127.s010.tif]

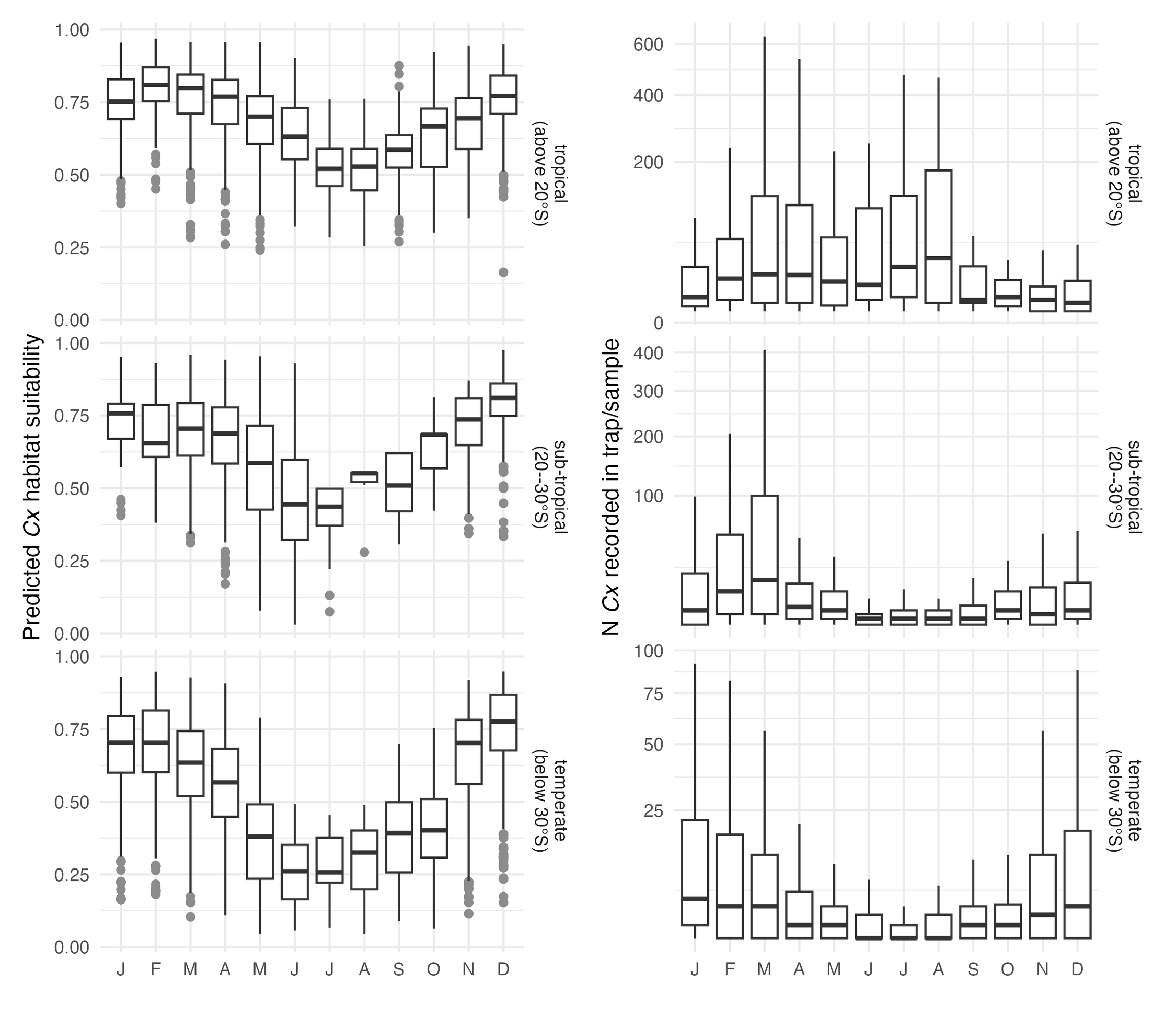

Supplement: S10 Fig — A) habitat suitability predictions at raster cells corresponding to Cx cohort occurrence data, and, B) independent comparison of maximum abundance per cell x month from the subset of those cells where the occurrence data from trap collections had an abundance attribute. Outliers (extreme high values) were omitted from B to emphasise the core 95% of the distribution. (TIF) [file pntd.0014127.s011.tif]

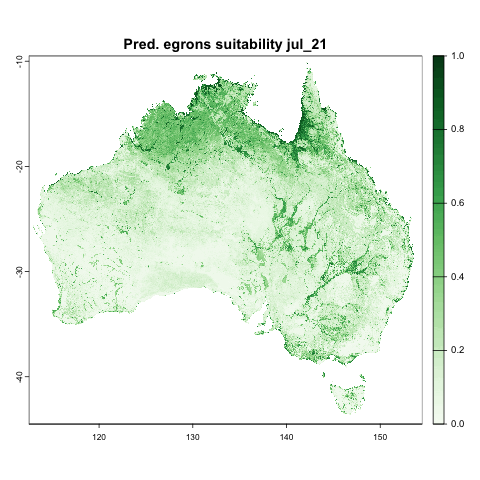

Supplement: S2 File — Monthly predictions of habitat suitability for ardeid cohort to July 2021–June 2023 available at this link. Base maps obtained from Australian Bureau of Statistics (CC BY 4.0) https://www.abs.gov.au/statistics/standards/australian-statistical-geography-standard-asgs-edition-3/jul2021-jun2026/access-and-downloads/digital-boundary-files. (GIF) [file pntd.0014127.s012.gif]

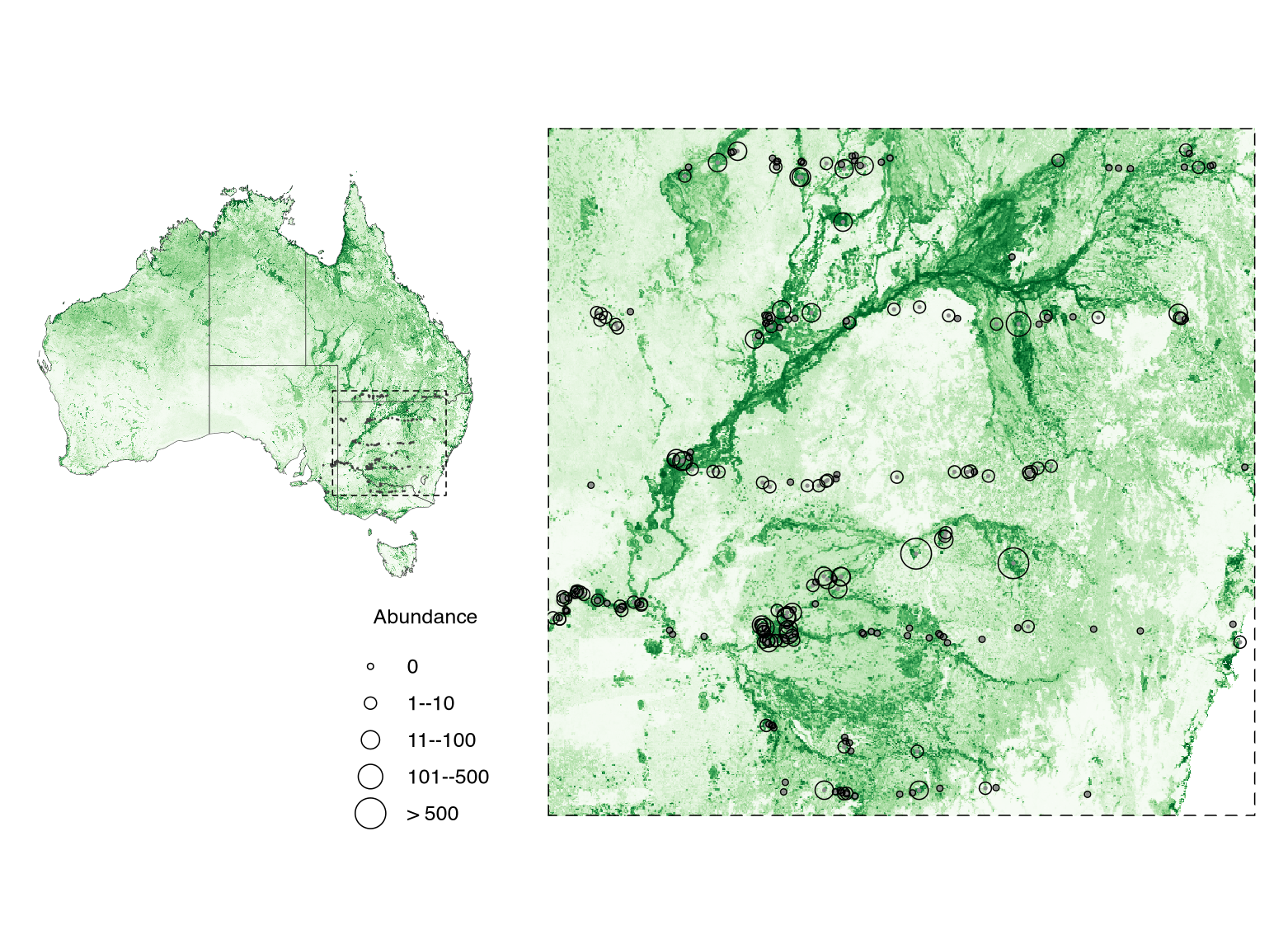

Supplement: S11 Fig — Grey dots indicate sightings of non-ardeid species and also indicate the flight path; black circles correspond to ardeid species sightings and are scaled by the recorded number of individuals. Base maps obtained from Australian Bureau of Statistics (CC BY 4.0) https://www.abs.gov.au/statistics/standards/australian-statistical-geography-standard-asgs-edition-3/jul2021-jun2026/access-and-downloads/digital-boundary-files. (TIF) [file pntd.0014127.s013.tif]

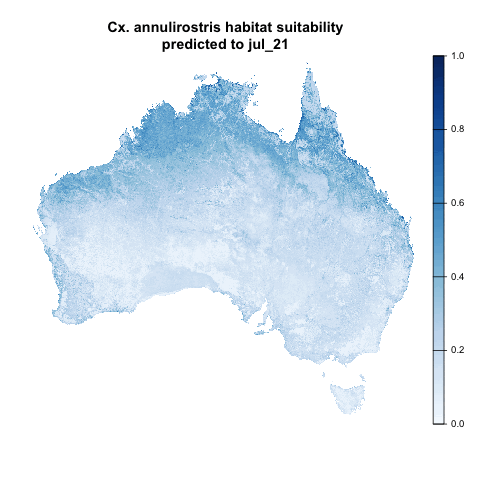

Supplement: S3 File — Base maps obtained from Australian Bureau of Statistics (CC BY 4.0) https://www.abs.gov.au/statistics/standards/australian-statistical-geography-standard-asgs-edition-3/jul2021-jun2026/access-and-downloads/digital-boundary-files. (GIF) [file pntd.0014127.s014.gif]
