# Supplementary material for: A time-varying geospatial model of habitat suitability for Japanese encephalitis virus vectors and vertebrate hosts in Australia
Source: PLoS Negl Trop Dis. 2026 Mar 20;20(3):e0014127. doi: 10.1371/journal.pntd.0014127 (PMC13035343; doi:10.1371/journal.pntd.0014127)
Supplement: Attachment — Submitted filename: response_to_reviewers.pdf [file pntd.0014127.s016.pdf]

Response to Comments

Line #	Comment	Response and Changes
**General**		
—	**R3** Both the "Author summary" and "Introduction" mention that an unprecedented outbreak happened in 2021/2022, but why this particular year was exceptional? For example, were climatic conditions usually favorable for vectors, hosts, or human-mosquito contact, or could this have occurred largely by chance? How did climatic conditions in summers prior to 2021 or after 2023 compare with those in 2021/2022? In addition, can JE transmission over-winter in Australia? That is to say, is reintroduction of cases required each year? Clarifying this point may help explain the large interannual variability in the reported human cases.	We have added references to the frequency of La Niña climate events to the "Author summary", "Introduction" (L15–19) and "Discussion" (L612–614, L621–622, L641–645) to emphasise that the events of the 2021–2023 outbreak are likely attributable to rainfall and inundations associated with that phenomenon. Those events explain hyper-abundance of breeding habitats, though they wouldn't influence over-wintering success *per se*, which is expected to be possible even if the mechanisms are still unclear (L678–690).
—	**R3** To help better interpret the results, it may be useful to add maps showing the geography, population distribution, summary climate variables (e.g., annual mean day or night temperature, EVI, rainfall as shown in Table 1), and land cover maps in the main text or supplementary materials.	Thanks for these suggestions. We have prepared additional Supplementary plots; a new map (S1 Fig) to summarise key geographic features mentioned in the text and new Fig S5 to display covariate data for those variables where some variation is discernible at that scale.

Line #	Comment	Response and Changes
—	**R3** Since multiple data sources were combined to develop the geospatial models for vectors and hosts, please clarify how consistency in data quality across sources was ensured. Were sensitivity analyses performed by excluding one or multiple data sources at a time? If so, did this substantially affect the results?	This is a very reasonable question. Our exclusion, filtering, and down-sampling approach is outlined in detail in the methods. We expected the data we obtained from state and territory surveillance teams, and the literature to be of the highest quality, and we restricted the GBIF and ALA data using the filters available. We didn't strictly need to include data from ALA and GBIF, but since the position of the filtered records from those sources don't stand out as implausible alongside the top quality data, and since other published studies have exclusively used the ALA and GBIF records, we wanted to include them to somehow connect to those studies. However, we did update the mosquito vector bias plot in the supplementary materials (now Fig S8) to show how the surveillance data that we obtained substantially reduced the bias inherent in the GBIF and ALA data - at least as measured by accessibility to areas of large population. Lastly, though invisibly to a reader, over the course of our project we have run the models scores of times for motives ranging from updated datasets to unforced programming errors and from that experience we are confident that their inclusion does not affect or degrade the results.
—	**R1** The manuscript is robust and presents analyses leading to a highly relevant discussion. The authors grounded their work in the creation of dynamic geospatial habitat suitability models for Japanese Encephalitis Virus (JEV) vectors and vertebrate hosts in Australia, leveraging the most comprehensive national datasets available. The model effectively visualizes the background conditions that can drive JEV spillover to humans, which has proven utility in public health settings. The research emphasizes its translational value by stating that the models and insights should be utilized to improve surveillance and preparedness for future outbreaks. The proposal to combine their fundamental habitat representation with comprehensive infection data (in all hosts) is particularly valuable. This nuanced understanding of transmission risk is essential for guiding appropriate policies under a One Health framework.	Thank you for the positive comments. We invested enormous effort to create a more complete national database of vectors in particular, though it is still not comprehensive.

Line #	Comment	Response and Changes
—	**R1** The primary strength of this work is the innovative methodology: the development of dynamic geospatial habitat suitability models for JEV. This represents a significant and novel contribution, moving beyond static models to illuminate the temporal and spatial conditions that drive viral spillover events. The overall execution of the analysis is meticulous, demonstrating a high level of scholarship by the authors. The importance of the study is undeniable, as its findings have direct utility in One Health contexts and in political decision-making. The ability to identify and predict high-risk transmission zones is fundamental for enhancing surveillance preparedness and preventing future outbreaks. The study is a well-executed piece of research that significantly advances the field's predictive capacity for JEV.	Thank you.
—	**R1** The manuscript's discussion and conclusions are solid and well-founded. The core strength lies in the fact that the conclusions are consistently backed by the presented data, effectively reinforcing the credibility of the findings and avoiding overstatement. Furthermore, the limitations of the analysis are clearly and appropriately described, which offers the necessary context for interpreting the study's scope. The authors have effectively discussed how these data are useful for advancing the field's understanding of the subject matter. Importantly, the relevance of the study to public health is explicitly and pertinently addressed, demonstrating the valuable practical impact of this research.	Thank you.

Line #	Comment	Response and Changes
—	**R2** The manuscript is well written, with clearly stated objectives, a sound study design, and well-presented use of epidemiological models and results. However, whether the current model can reliably predict future, similar outbreaks remain to be confirmed. Although the model's predictions correlate with the 2022 JEV outbreak, its ability to predict other similar outbreaks still needs to be validated. Additionally, while the findings suggest that the Australian ecosystem is suitable for both vectors and hosts, the precise role this plays in linking the presence of the virus to these species has not been fully defined.	It is true that we have not validated our models against detections from past outbreaks of related flaviviruses, nor against detections of JEV from after the 2021–2023 outbreak. These would be interesting and valuable extensions but we feel that they fall outside the scope of this paper
**Abstract**		
	**R3** The "Great dividing range" was mentioned but does not appear to be labelled or shown in any figure. It could be added to an existing map.	A map including geographic features mentioned in the text has been added as Fig S1.
**Introduction**		
—	**R3** It might be helpful to briefly describe some ecology and life history of the Culex genus, particularly the habitats preferred by their aquatic stages (i.e., artificial containers versus natural water bodies). This context could assist readers in interpreting the exposure-response relationships shown on the right side of Figs 1 and 2.	Thanks for this suggestion. We have moved some ecological information from the Methods into the paragraph about *Culex* vectors in the Introduction (L45–51), bolstering it with some additional reference to the larval habitats.

Line #	Comment	Response and Changes
L68	**R3** Pigs were excluded from the study due to the lack of recent data. However, L62 mentioned that tens of millions of feral pigs are widely distributed across half of the Australian mainland. If this is the case, could leaving out pigs in the host modelling part introduce bias? If historical pig distribution data exist, even if imperfect, would it be feasible to conduct a sensitivity analysis including pigs?	This is an important question, though note that the underlying *data* which contributed to those models are themselves not readily available, and the work to obtain and harmonise those datasets would have been immense (which may explain why the recent release of an updated model from ABARES/CSIRO has taken so long). Over the course of the project we explored the use of the existing *models* of feral pig distribution in the development of what became our combined and rescaled suitability map (Figure 3). Since the main thrust of our submission was bringing together major primary datasets to build host and vector habitat models we decided to defer the integration of feral and domestic pig distribution models to a linked second piece of work (Harrison et al; see cover letter). It fits much better there as that piece combines the outputs from the current work and those pig distribution models all as inputs of equal standing (best available distribution models) into models of the potential and realised pathogen niche. We appreciate that this information about the forthcoming paper wouldn't reduce the desire that a reader of *this* work may have to gain more insight about evidence for the importance of feral pigs, but we do set out our rationale in the Introduction.
—	**R3** Are there other local animal species, beyond pigs and ardeid birds, that may play a role in the natural transmission of JEV in Australia?	There are other species for which laboratory infection experiments or field survey have indicated potential competence as hosts. Also, other groups like marsupials have been shown to be a preferred blood meal of *Cx annulirostris* in at least one study. We have added references to the Introduction to communicate this to readers.
**Methods**		

Line #	Comment	Response and Changes
—	**R1** This study addresses a highly relevant public health issue, and its objectives are notably well-articulated, establishing a clear and empirically testable hypothesis. The selected methodological design is entirely appropriate for these objectives. Crucially, the study population is precisely described and pertinent to the research question, and the sample size is sufficient to ensure adequate statistical power. The statistical analyses employed are rigorous and correct, providing firm support for the conclusions drawn. Furthermore, the manuscript confirms full compliance with all ethical and regulatory requirements, with no concerns identified in this regard. Overall, the work demonstrates strong foundational rigor in its design, population definition, and statistical approach, making it a robust contribution.	Thank you.
—	**R2** The objectives are clearly defined and use of different databases available and used for analysis are comprehensive. The use of publicly available data to define the cohorts are described well in the manuscript. The statistical models using logistic and boosted regressions for occurrence for mosquito and ardeid host species are clearly defined. The combined model for host and vector are well described.	Thank you.
L118–120	**R3** Please clarify the composition of *Culex* species. What proportion of the total *Culex* population is represented by each species, especially *Cx. annulirostris*, and does this vary geographically? A supplementary bar plot showing species composition by year or location, or coloring the dots in Fig 1A by species, could help justify treating *Culex* as a single group.	Thanks for the suggestions about how to rectify this deficiency. Since Fig 1A is so small we have opted to include an additional Supplementary plot (Fig S2) with the species by species occurrence data. We added a corresponding supplementary figure for the ardeid species (Fig S3).
L138	**R3** Table 2 is cited before Table 1. The order of these two tables may need to be reversed.	Thank you for picking this up. We have removed the earlier reference to Table 2 so the existing order is appropriate.

Line #	Comment	Response and Changes
L143	**R3** The rational for using *Cx. annulirostris* in the sensitivity analyses is clear. However, why *N. caledonicus* selected?	On reflection, we scarcely mentioned the Nankeen night heron (*N. caledonicus*) model in the text so we have decided to remove it from the Supplement. There is some evidence that highlights *N. caledonicus*, but it is not highlighted with the same weight of published opinion about its role.
Table 1	**R3** Please clarify the meaning of " " in the Sampling method column.	The text in question was meant to indicate 'as above' or the continuation of the previously specified response. Since there were many rows and few different methods we moved the sampling details into the text in this iteration.
Table 1	**R3** Please add references in brackets for rows under the "Public datasets" and "Published literature" subsections.	These sources have now been linked to their formal citation in the bibliography. The records that were attributed to the VectorMap database – a project of the Smithsonian Institute's Walter Reed Biosystematics Unit – can no longer be accessed on the internet due to the cessation of funding for the institution in 2025. I have identified the linked source publication (Frances et al 2009) and moved those records accordingly.
Table 2	**R3** Table 2: Please clarify the observation protocols "500m area search", "stationary", "travelling", and "2ha-20min search".	A brief definition of each protocol - and the features common to the set - has been added to the Methods at L243–250.
Table 3	**R3** It might be better to switch the order of the climatic and land cover dataset to match their orders in the main text. Moreover, using consistent category titles as in the main text (i.e., "static land cover variables" and "temporally dynamic environmental variables") and matching the variable names with those used in Figs 1 and 2 would also improve clarity.	Thank you for these suggestions. The sections have been renamed and the order changed to match the text subheadings.
L306–321	**R3** Were these data processing steps applied to public datasets and published literature as well? If not, how to account for reporting bias in these two sources? This relates to the earlier comment regarding harmonization of data quality prior to merging datasets.	Yes, the target-group background records of Animalia was the bias correction method for the entire *Culex* dataset, which were all treated as one set degraded to occurrence data as though they were presence only (L321–323).

**Line #**	**Comment**	**Response and Changes**
L341–363	**R3** Were pseudo-absence locations needed in the model? Was a single model fitted across all months, or were separate models fitted to each month?	Target-group background locations are often used synonymously with pseudo-absence locations; the former terminology describes what these data were whereas the latter conjectures what they may or may not be (an absence). The background (quadrature) points play the same role in a model in either case - as a contrast set of points with which to test discrimination of the environmental space associated with the species occurrences from that associated with the target group or psuedo-absences. We did need to use these points to obtain a model fit to produce a continental scale map output. As we explained in the paragraph at L321–323, the trap data in surveillance datasets and published datasets could be interpreted as presence-absence data. However, the criteria that drive surveillance activities mean that traps were not deployed broadly across geographic and environmental space.
L339–393	**R3** Please explain why different models were used for vectors/hosts?	In principle we could have run both of these models using boosted regression trees however due to the pattern of spatial bias in the mosquito trapping data our intuition was that the ease of control over regularization offered by existing packages implementing maxent / maxnet algorithms was a good fit for the vector modelling problem. Also, the models took several hours to run so the convenience features of those packages were important to getting the work done. Text has been added to the Methods (L390–394) to that effect.
L406–409	**R3** A map illustrating the spatial blocks used in the analysis may be helpful.	A map of the spatial blocks has been added to the supplementary material as Fig S7.
L419	**R3** Please clarify the rational for "using the predictions for February 2022"?	We now note that February 2022 was chosen because it corresponded with or immediately preceded a period of active detections across most Australian jurisdictions (L462–464).
**Results**		

Line #	Comment	Response and Changes
—	**R1** The methodological evaluation confirms that the presented analysis rigorously matches the pre-established analytical plan, which is essential for guaranteeing the study's coherence and internal validity. Moreover, the results are presented in an exceptionally clear and complete manner, facilitating straightforward interpretation of the key findings. The quality of the supporting materials is also high; the figures, including tables and images, are of high quality and entirely sufficient for clarity, significantly enhancing the comprehension of the complex data. This high standard of reporting allows the reader to easily follow the analytical path from raw data to reported results, establishing confidence in the findings.	Thank you.
—	**R2** The results are presented well and clearly. The figures need some work, as fig 2 h and i, have the names cropped.	Thank you - those figure labels have now been aligned on both figures 1 and 2.
L448	**R3** "2023 1a" at the end of the line should be Fig. 1a?	Thank you, this error has been corrected.
L530–538	**R3** Would it be appropriate to incorporate human population density into the combined model to better represent JE risk? Additionally, what are the possible reasons for the absence of human or pig cases in northern Australia near Karumba, although it has high predicted habitat suitability index as shown in Fig 3?	This is an excellent point. JE risk would be better evaluated with respect to human population and more comprehensive surveillance data across a range of species, including humans. That is beyond the scope of this paper, and we address it in our linked manuscript (Harrison et al; see cover letter) which will be submitted very soon.
**Discussion**		
—	**R2** The discussion, including the limitations of the study, highlights the model's potential applicability to other flaviviruses. While the authors focus primarily on JEV in this manuscript, they also note the model's relevance to other flaviviruses present in Australia. However, the connection between the outcomes of this model and other endemic flaviviruses in Australia still needs to be clearly established.	We agree. We feel we were appropriately circumspect in noting the possibility that the models that we produced may indicate potential habitat suitability for other flaviviruses in Australia. Further work including is required to test these ideas including studies capable of at least conceptually disentangling the co-circulation of this flavivirus group.
