# Supplementary material for: A time-varying geospatial model of habitat suitability for Japanese encephalitis virus vectors and vertebrate hosts in Australia
Source: PLoS Negl Trop Dis. 2026 Mar 20;20(3):e0014127. doi: 10.1371/journal.pntd.0014127 (PMC13035343; doi:10.1371/journal.pntd.0014127)
Supplement: Attachment — Submitted filename: Plos_NTDs_JE_Australia.docx [file pntd.0014127.s015.docx]

This manuscript develops two geospatial models with comprehensive datasets on the vectors and hosts for JE in Australia. Overall, the method framework is sound, the analyses appear carefully conducted, and the manuscript is clearly written and easy to follow.

General comments:

- Both the “Author summary” and “Introduction” mention that an unprecedented outbreak happened in 2021/2022, but why this particular year was exceptional? For example, were climatic conditions usually favorable for vectors, hosts, or human-mosquito contact, or could this have occurred largely by chance? How did climatic conditions in summers prior to 2021 or after 2023 compare with those in 2021/2022? In addition, can JE transmission overwinter in Australia? That is to say, is reintroduction of cases required each year? Clarifying this point may help explain the large interannual variability in the reported human cases.
- To help better interpret the results, it may be useful to add maps showing the geography, population distribution, summary climate variables (e.g., annual mean day or night temperature, EVI, rainfall as shown in Table 1), and land cover maps in the main text or supplementary materials.
- Since multiple data sources were combined to develop the geospatial models for vectors and hosts, please clarify how consistency in data quality across sources was ensured. Were sensitivity analyses performed by excluding one or multiple data sources at a time? If so, did this substantially affect the results?

Abstract

- The “Great dividing range” was mentioned but does not appear to be labelled or shown in any figure. It could be added to an existing map.

Introduction

- It might be helpful to briefly describe some ecology and life history of the *Culex* genus, particularly the habitats preferred by their aquatic stages (i.e., artificial containers versus natural water bodies). This context could assist readers in interpreting the exposure-response relationships shown on the right side of Figs 1 and 2.
- L 68: Pigs were excluded from the study due to the lack of recent data. However, L62 mentioned that tens of millions of feral pigs are widely distributed across half of the Australian mainland. If this is the case, could leaving out pigs in the host modelling part introduce bias? If historical pig distribution data exist, even if imperfect, would it be feasible to conduct a sensitivity analysis including pigs?
- Are there other local animal species, beyond pigs and ardeid birds, that may play a role in the natural transmission of JEV in Australia?

Methods

- L118-120: Please clarify the composition of *Culex* species. What proportion of the total *Culex* population is represented by each species, especially *Cx. annulirostris*, and does this vary geographically? A supplementary bar plot showing species composition by year or location, or coloring the dots in Fig 1A by species, could help justify treating *Culex* as a single group.
- L138: Table 2 is cited before Table 1. The order of these two tables may need to be reversed.
- L143: The rational for using *Cx. annulirostris* in the sensitivity analyses is clear. However, why *N. caledonicus* selected?
- Table 1: Please clarify the meaning of “” in the Sampling method column.
- Table 1: Please add references in brackets for rows under the “Public datasets” and “Published literature” subsections.
- Table 2: Please clarify the observation protocols “500m area search”, “stationary”, “travelling”, and “2ha-20min search”.
- Table 3: It might be better to switch the order of the climatic and land cover dataset to match their orders in the main text. Moreover, using consistent category titles as in the main text (i.e., “static land cover variables” and “temporally dynamic environmental variables”) and matching the variable names with those used in Figs 1 and 2 would also improve clarity.
- L306-321: Were these data processing steps applied to public datasets and published literature as well? If not, how to account for reporting bias in these two sources? This relates to the earlier comment regarding harmonization of data quality prior to merging datasets.
- L341-363: Were pseudo-absence locations needed in the model? Was a single model fitted across all months, or were separate models fitted to each month?
- L341-363: Should host availability be accounted in the vector model? More specifically, should the host model be fitted first, and then use the results of it as inputs for the vector model?
- L339-393: Please explain why different models were used for vectors/hosts?
- L406-409: A map illustrating the spatial blocks used in the analysis may be helpful.
- L419: Please clarify the rational for “using the predictions for February 2022”?

Results

L448: “2023 1a” at the end of the line should be Fig. 1a?

L530-538: Would it be appropriate to incorporate human population density into the combined model to better represent JE risk? Additionally, what are the possible reasons for the absence of human or pig cases in northern Australia near Karumba, although it has high predicted habitat suitability index as shown in Fig 3?
